# Mannose phosphate isomerase deficiency‐congenital disorder of glycosylation (MPI‐CDG) with cerebral venous sinus thrombosis as first and only presenting symptom: A rare but treatable cause of thrombophilia

**DOI:** 10.1002/jmd2.12149

**Published:** 2020-08-06

**Authors:** Chris Mühlhausen, Lisa Henneke, Lars Schlotawa, Daniel Behme, Marianne Grüneberg, Jutta Gärtner, Thorsten Marquardt

**Affiliations:** ^1^ Department of Pediatrics and Adolescent Medicine University Medical Centre Göttingen Göttingen Germany; ^2^ Department of Neuroradiology University Medical Centre Göttingen Göttingen Germany; ^3^ Department of General Paediatrics University of Münster Münster Germany

**Keywords:** antithrombin deficiency, cerebral venous sinus thrombosis, coagulation disorder, congenital disorder of glycosylation, MPI‐CDG, thrombophilia

## Abstract

Mannose phosphate isomerase deficiency‐congenital disorder of glycosylation (MPI‐CDG; formerly named CDG type 1b) is characterized by the clinical triad of hepatopathy, protein‐losing enteropathy, and hyperinsulinemic hypoglycemia in combination with coagulation disorder (thrombophilia, depletion of antithrombin, proteins C and S, factor XI). In the majority of patients, MPI‐CDG manifests during early infancy or childhood. Here, we present a 15‐year‐old female patient with unremarkable medical history suffering from acute cerebral venous sinus thrombosis necessitating interventional thrombectomy and neurosurgical decompression. Diagnostic work‐up of thrombophilia revealed deficiency of antithrombin (AT), proteins C and S, and factor XI. Detailed evaluation identified MPI‐CDG as the underlying cause of disease. After initiation of mannose therapy, coagulation parameters normalized. The girl fully recovered without any neurologic sequelae, and remains free of further thrombotic events or any other clinical and laboratory abnormalities on follow‐up 1 year after start of mannose treatment. In conclusion, we here present the significant case of MPI‐CDG with a severe cerebral venous sinus thrombosis as the first and only symptom of the disease. In light of the high frequency of AT deficiency on one hand, and the excellent treatability of MPI‐CDG on the other hand, CDG screening should be included as a routine analysis in all patients presenting with unexplained coagulation disorder, especially when comprising AT deficiency.

**SYNOPSIS**

This presentation of the first MPI‐CDG patient manifesting with severe cerebral venous sinus thrombosis as the first and only symptom supports the recommendation that CDG screening should be included into the routine diagnostic work‐up in all patients presenting with unexplained coagulation disorder, especially when comprising AT deficiency.

## 1 INTRODUCTION

Congenital disorders of glycosylation (CDG) are a group of inherited multisystem disorders with a broad range of symptoms ranging from coagulation abnormalities to neuropathy, ataxia, dysmorphic features, skeletal deformities, and severe psychomotor retardation and intellectual disability. A biochemical hallmark of most CDG syndromes is the hypoglycosylation of newly synthesized proteins, leading to either truncated or completely missing carbohydrate side chains of glycoproteins such as transferrin, antithrombin (AT), protein C, protein S, factor XI and many others.^1^, ^2^ Mannose phosphate isomerase deficiency‐CDG (OMIM #602579, MPI‐CDG, formerly designated CDG type 1b) is caused by the deficiency of mannose phosphate isomerase (MPI; OMIM #154550; EC 5.3.1.8), which converts fructose‐6‐phosphate to mannose‐6‐phosphate,^3^ an essential substrate for the initial steps of protein glycosylation in the endoplasmic reticulum.^1^ Affected patients display a characteristic CDG type 1 transferrin isoelectric focusing pattern. The clinical presentation primarily comprises hepatic and gastrointestinal symptoms including protein‐losing enteropathy, failure to thrive, hepatic fibrosis, as well as hyperinsulinemic hypoglycemia and coagulation disorder (depletion of AT, proteins C and S, factor XI) with frequent thromboses, all of which have a considerable degree of clinical heterogeneity. Unlike in other CDG types, no primary neurological abnormalities have been described in MPI‐CDG patients.^4^, ^5^, ^6^ The oral administration of mannose has been established as an efficient therapy, as cells efficiently take up mannose via a specific mannose transporter and subsequently convert it to mannose‐6‐phosphate, thus bypassing the metabolic block.^1^, ^3^, ^5^, ^7^, ^8^ Here, we present the first case of a patient with a cerebral venous sinus thrombosis as the first and only presenting symptom of MPI‐CDG.

## 2 CASE REPORT

A 15‐year‐old girl was admitted to the emergency department due to strongest headache, ataxia, vomiting, and progressive somnolence. The complaints had started 24 hours prior to admission, following a three‐week period of abnormal fatigue. Four weeks before admission, the patient had started to take an oral contraceptive for family planning purposes, any other medication was denied.

**FIGURE 1:**
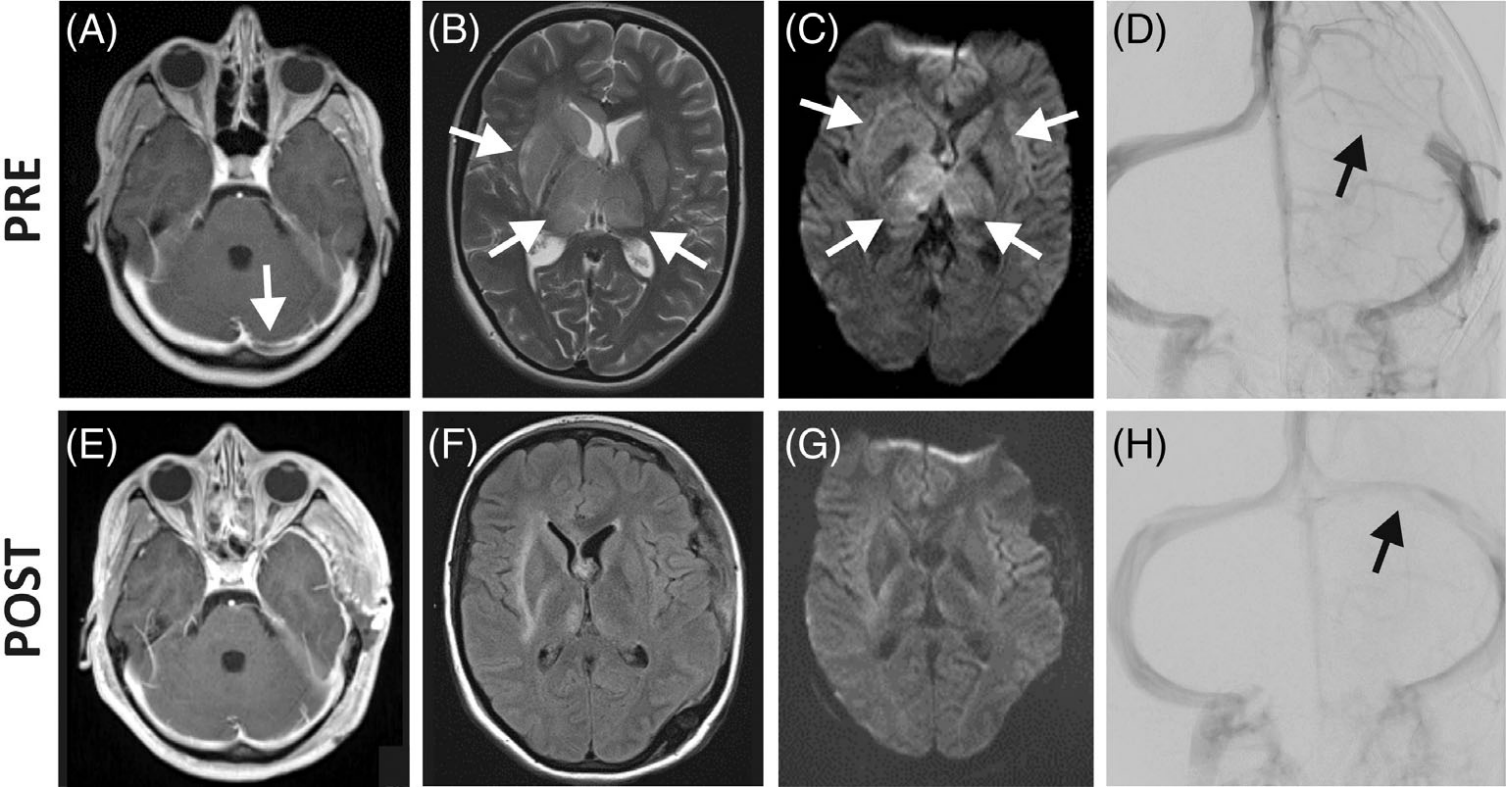
Neuroradiologic findings. MRI, A‐C, E‐F, of the brain and angiographic, D, H, findings immediately before (pre) and directly, H, or 2 weeks, E‐G, after (post) interventional thrombectomy. A, E, Contrast medium T1‐weighted MRI revealed occlusion of the left transversus sinus (arrow) which was restored post‐interventionally. T2‐/FLAIR, B, F; and DWI‐weighted, C, G; MRI displayed alterations suggestive for bilateral congestive infarction of the basal ganglia and thalami before thrombectomy (B, C, arrows), which ameliorated 2 weeks after the intervention, F, G. Contrast‐mediated cerebral angiography (D, H; both AP projection) illustrates blockage of the left transversus sinus (D; arrows) and its recanalization directly after interventional aspiration thrombectomy (H)

Progressive somnolence necessitated intubation and assisted ventilation. The initial cCT and successive cMRI scan revealed an extensive cerebral venous sinus thrombosis (CVT) of the straight sinus, left transverse sinus, and the occipital part of the superior sagittal sinus with subsequent congestive infarction of the basal ganglia and thalami (Figure 1A‐C). During the immediate angiography, a 6 cm venous thrombus could be interventionally removed by aspiration. Mechanical thrombectomy was carried out under general anesthesia. A diagnostic catheter was placed to the left internal carotid artery via the right femoral artery. A diagnostic run was performed confirming CVT of the internal cerebral veins, the vein of Galen, the straight sinus and the proximal part of the left transverse sinus (Figure 1D). A therapeutic catheter was introduced via the right femoral vein to the left jugular bulb, and advanced to the level of the thrombotic occlusion of the left transverse sinus. Continued aspiration was carried out for 20 minutes within the transverse sinus and secondary within the straight sinus. After aspiration thrombectomy, flow was restored within the transverse sinus (Figure 1H). However, progressive congestive brain edema required implantation of an intracranial pressure monitor and subsequently an external cerebrospinal fluid drainage and surgical decompression by partial removal of calvarium bone. Intracranial pressure improved continuously, so that drainages and monitor could be removed after 2 weeks. After cessation of ventilation and anesthesia, the girl initially showed a left‐sided hemiparesis and a motoric aphasia. She was able to communicate by movements with her eyes and head. A control cMRI 2 weeks post intervention revealed amelioration of basal ganglia congestive infarction alterations (Figure 1F,G). During the following rehabilitation, she fully recovered, and on examination 5 months later, she showed no neurological sequelae. The girl returned to school with normal performance.

Immediately after diagnosis of cerebral venous sinus thrombosis, therapy with unfractionated heparin was initiated and changed to low molecular weight heparin 2 weeks later. After 5 months, anticoagulative treatment was shifted to phenprocoumon. The oral contraceptive had been discontinued instantly after initial hospital admission.

**FIGURE 2:**
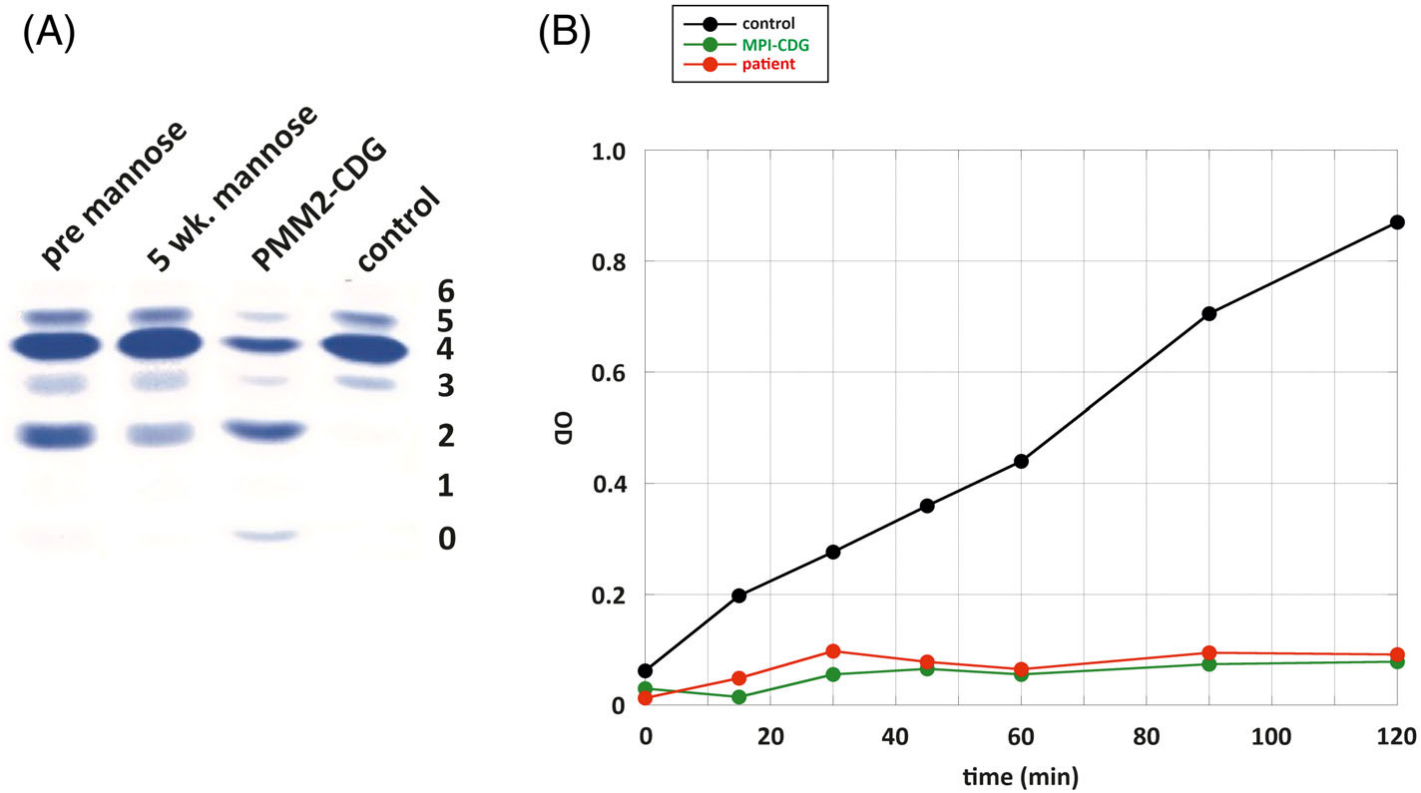
Glycosylation analysis and phosphomannose isomerase activity assay. A, Transferrin isoelectric focusing analysis of the patient (pre mannose) reveals CDG type I glycosylation pattern. PMM2‐CDG patient and healthy (control) sera were used as negative and positive controls, respectively. After 5 weeks of mannose treatment, the patient showed vanishing of asialo‐ and decrease of disialo‐transferrin. B, MPI activity assay in leukocytes of patient (red) reveals absence of MPI activity comparable to a confirmed MPI‐CDG patient (green), in contrast to normal activity of a healthy control (black)

An extensive diagnostic work‐up regarding thrombophilia including lipoprotein a, prothrombin G20210A and factor V Leiden mutations revealed no abnormal findings. Methylenetetrahydrofolate reductase (*MTHFR*) gene analysis showed the thermolabile variant c.655C>T (p.Ala222Val) in heterozygous state, but homocysteine in plasma was repeatedly normal. Proteins C, S, and factor XI were abnormal. Of note, AT was persistently low. Subsequently, SERPINC1/AT gene analysis was performed but revealed no abnormal findings. Further evaluation of causes of reduced AT activity revealed an abnormal glycosylation pattern in transferrin isoelectric focusing analysis, indicating a congenital disorder of glycosylation (CDG) type 1 (Figure 2A). Enzymatic analysis of mannose phosphate isomerase (MPI) in leukocytes displayed absent activity, proving MPI‐CDG (Figure 2B). This was confirmed by *MPI* gene analysis showing compound heterozygous occurrence of the variants c.655C>T/p.Arg219Trp and c.1178G>C/p.Gly393Ala. The patient's mother carried the variant c.655C>T/p.Arg219Trp in heterozygous state and showed c.1178G as wildtype allele; DNA of the father, however, was not obtainable. Both variants have not been reported in the literature yet but showed an allele frequency of 0.002% and 0.02%, respectively, according to the dbSNP database (entries rs138891630 and rs201815588). They were therefore considered pathogenic. An extensive evaluation of the preceding medical history did not reveal any gastrointestinal, hepatic or endocrine symptoms during infancy, childhood and adolescence (Table 1), as they have been frequently reported in other MPI‐CDG patients.

**TABLE 1:** Patient's history and results regarding frequent MPI‐CDG symptoms

Parameter	Result
Previous history of enteropathy and/or chronic diarrhea?	No
Previous history of failure to thrive?	No
Previous history of clinically overt liver disease?	No
Previous history of events suspicious for hypoglycemia?	No
Previous history of clinical signs of thrombosis?	No
Blood count	On admission normal; outside acute ICU period repeatedly normal leukocyte and thrombocyte count, occasionally slightly reduced hemoglobin, especially during menstruation (median 9.5 g/dL, normal range 11.5‐15.0)
Albumin (normal range 3.5‐4.9 g/dL)	On admission 3.6 g/dL, outside acute ICU period repeatedly normal
AST (normal range 17‐33 U/L)	On admission 21 U/L, outside acute ICU period repeatedly normal
ALT (normal range 8‐24 U/L)	On admission 12 U/L, outside acute ICU period repeatedly normal
Alkaline phosphatase (normal range 40‐150 U/L)	On admission 63 U/L, outside acute ICU period repeatedly normal
γGT (normal range 7‐21 U/L)	On admission 7 U/L, outside acute ICU period repeatedly normal
TSH (normal range 0.75‐4.94 mIU/L)	On admission 3.36 mIU/L, outside acute ICU period 1.26 mIU/L
fT4 (normal range 7.0‐14.8 ng/L)	On admission 7.6 ng/L, outside acute ICU period 7.0 ng/L
Liver ultrasound	On admission: normal size of liver and spleen, normal liver echotexture, no liver nodules, normal liver and spleen vasculature and perfusion

**FIGURE 3:**
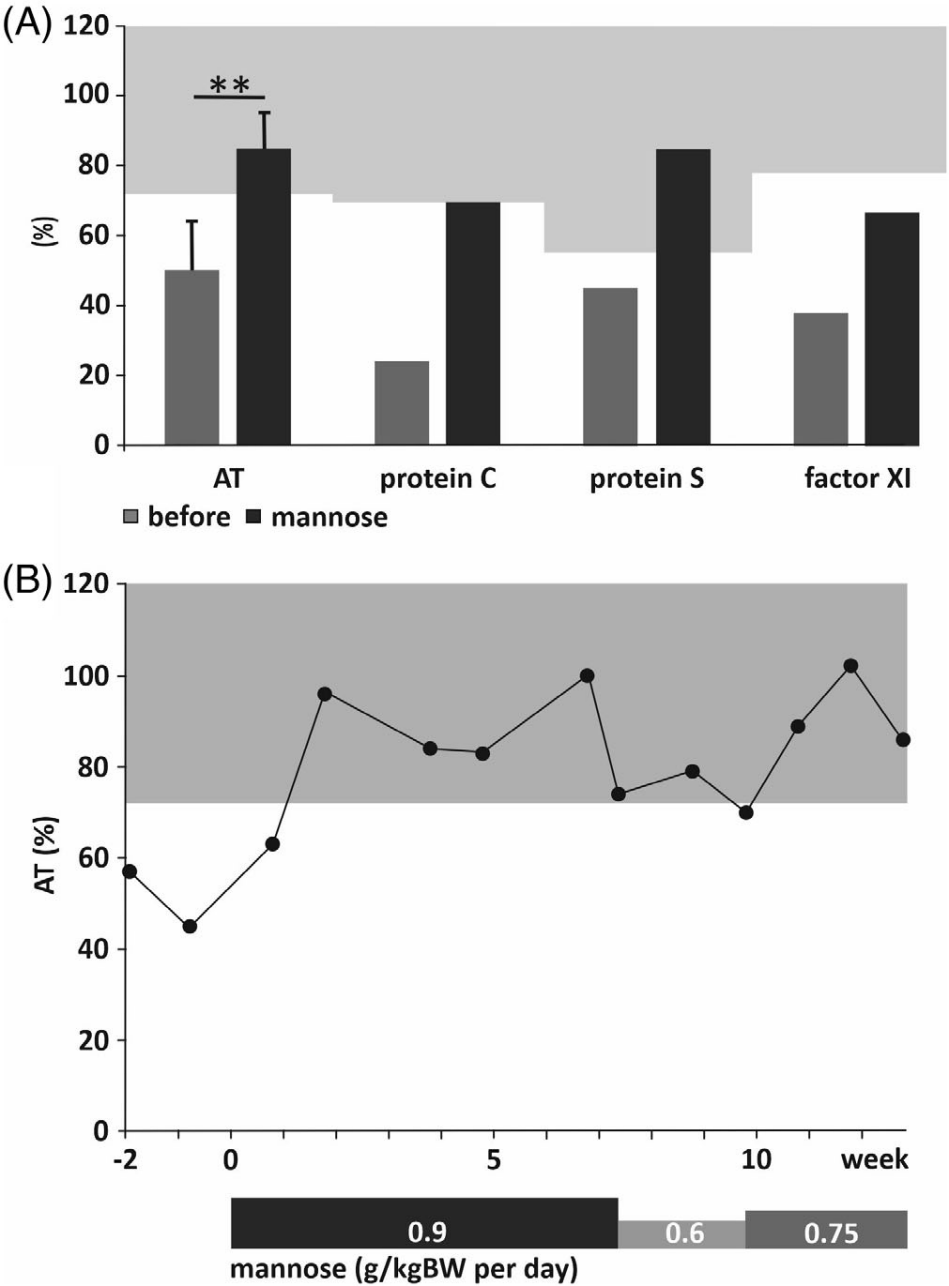
Activities of coagulation system glycoproteins before and after initiation of mannose therapy. A, Activities of AT, protein C, protein S and factor XI before (dark‐grey bars) and during (black bars) mannose therapy. Error bars indicate SD for AT measurements (n = 22 before and n = 18 during therapy). Number of measurements of protein C, protein S and factor XI were 1‐2, respectively, thus no error bars or statistics were calculated. Light grey areas indicate normal ranges of the respective glycoprotein activities. Level of significance was determined by unpaired two‐sided Student's *t* test; **P < .001. B, Time course of AT activity 2 weeks before start and during mannose therapy, dosing as indicated below. Light grey area indicates normal range of AT activity

Following the diagnosis, a therapy with oral supplementation of D‐mannose was initiated at a dose of 0.9 g/kgBW per day in 3 to 4 divided doses. AT, protein C, protein S and factor XI activities almost or fully normalized within 2 weeks (Figure 3), and phenprocoumon was discontinued. However, the patient suffered from loose stools and independently reduced the dose to 0.6 g/kgBW per day. Under this regimen, AT activity decreased again, so that the mannose dose was stepwise increased to 0.75 g/kgBW per day divided into 5 to 6 doses. On this mannose dose, AT normalized again (Figure 3B), without any further gastroenterologic complaints.

## 3 DISCUSSION

MPI‐CDG is a rare inherited metabolic disease with unknown prevalence. To date, about 35 patients have been described in the literature, most of them in single case reports. Clinical symptoms of published cases of MPI‐CDG patients have been summarized before^4^, ^6^ which enabled the description of a “classic triad” of symptoms, namely the combination of hepatic (liver fibrosis, hepatopathy), gastroenterologic (protein‐losing enteropathy) and endocrine (hyperinsulinemic hypoglycemia) symptoms in MPI‐CDG patients. In addition, 85% of patients exhibit a coagulopathy.^6^ The average age at onset of symptoms has been specified as 1.2 years, with only two patients manifesting during adolescence,^6^ and two patients identified during adulthood.^9^ The latter two patients have been described as asymptomatic, of note without any liver or intestinal disease. In contrast to liver, gastrointestinal and endocrine abnormalities, all MPI‐CDG patients described so far had normal intelligence and absence of any neurologic symptoms, albeit occurrence of a temporary developmental delay and muscular hypotonia in a few patients resolving within the first 2 years of life.^4^, ^6^ The coagulation disorder in MPI‐CDG patients comprises thrombotic events at various localizations including cerebral venous sinus,^10^ but has never been described as an isolated symptom of MPI‐CDG before.

In contrast to the different published cases, our patient presented with a severe and life threatening coagulation disorder and subsequent severe cerebral venous sinus thrombosis as the first and only presenting symptom of MPI‐CDG, with no other symptoms frequently found in MPI‐CDG patients. It might be speculated that the thrombophilia in this patient has been aggravated by the initiation of an oral contraceptive 4 weeks prior to hospital admission.

The administration of oral mannose constitutes a causal therapy of MPI‐CDG, resulting in amelioration or normalization of gastrointestinal, endocrine, and coagulation disease.^3^, ^8^, ^10^ However, hepatomegaly, hepatic fibrosis and liver dysfunction appear not to respond to mannose supplementation, thus liver transplantation has been suggested as a therapeutic option for selected patients.^11^ The published dosage of oral mannose varies from 600 to 1200 mg/kgBW per day.^4^, ^6^ Abdominal discomfort and diarrhea have been described as predominant side effects of oral mannose, usually resolving after dose adjustment or step‐wise introduction of mannose supplementation^4^, ^6^ as it has been observed in the present patient. It has been described that coagulation abnormalities resolve within weeks after beginning mannose supplementation, and no thromboses have been described in MPI‐CDG patients on mannose therapy.^3^, ^6^, ^8^ This is in line with observation of the patient presented here, in which AT activity normalized within 2 weeks after initiation of mannose treatment. On follow‐up 1 year after start of mannose administration, no further thrombotic events had occurred, nor any other hepatic, gastrointestinal, endocrine or neurologic symptoms.

In summary, we present the first MPI‐CDG patient with severe cerebral venous sinus thrombosis as first and only symptom of disease manifestation. A classical triad of symptoms has been described in MPI‐CDG patients, however with a notable degree of disease heterogeneity. The disease can be successfully treated by oral supplementation of a simple sugar, D‐mannose. Due to the observations presented here, more MPI‐CDG patients with varying phenotype and degree of disease may exist, and it may thus be speculated that MPI‐CDG is underdiagnosed especially when patients present without classical symptoms. This is in line with observations in adult patients with AT deficiency and chronic enteropathy misdiagnosed as inflammatory bowel disease.^12^ We therefore strongly suggest that carbohydrate‐deficient transferrin profile should be included into the evaluation of all patients with coagulation disorders of unknown origin, especially with unexplained thrombotic events or AT deficiency. This appears of special importance particularly in light of the excellent treatability of MPI‐CDG disease.

## 4 CONCLUSION

Here we present the first patient manifesting with severe cerebral venous sinus thrombosis as only symptom of MPI‐CDG disease. MPI‐CDG may be underdiagnosed and should not be missed in patients with coagulation defects. Thus, we strongly suggest to include carbohydrate‐deficient transferrin profile as a routine analysis in all patients presenting with unexplained coagulation disorder and AT deficiency given the excellent treatment options of MPI‐CDG disease.

## CONFLICT OF INTEREST

The authors declare that they have no conflicts of interest.

## AUTHOR CONTRIBUTIONS

Chris Mühlhausen was following the patient and designed, conceptualized and executed data analyses and interpretations, and drafted the manuscript. Lisa Henneke was following the patient, collected and analyzed the data, and reviewed and revised the manuscript. Lars Schlotawa was following the patient, designed and performed data collections and analyses, and reviewed and revised the manuscript. Daniel Behme analyzed neuroradiologic findings and data, and reviewed and revised the manuscript. Marianne Grüneberg carried out biochemical and genetic analyses, edited related figures, and reviewed and revised the manuscript. Jutta Gärtner conceptualized and executed data analyses and interpretations, and reviewed and revised the manuscript. Thorsten Marquardt performed biochemical and genetic analyses, designed and executed data analyses and interpretations, and reviewed and revised the manuscript. All authors read, reviewed, revised, and approved the final manuscript as submitted.

## ETHICS APPROVAL

All procedures described were carried out in accordance with The Code of Ethics of the World Medical Association (Declaration of Helsinki) and institutional guidelines.

## PATIENT CONSENT

Informed consent was obtained from the patient and her parents for inclusion of clinical descriptions, data, and images.
